# Supplementary material for: Wish-Granting Interventions Promote Positive Emotions in Both the Short and Long Term in Children with Critical Illnesses and Their Families
Source: Children (Basel). 2024 Dec 30;12(1):47. doi: 10.3390/children12010047 (PMC11763413; doi:10.3390/children12010047)
Supplement: Supplementary file 1 [file children-12-00047-s001.zip › children-3372718-supplementary.pdf]

Total Number of Responses as a Proportion of Total Wishes Granted in Last 5 Years

Note: Total wishes includes all wishes granted for children aged 3-17. This study examined children 13-17 only.

Table S1. Child survey responses.

**Affiliates**	**Number of responses**	**Total wishes granted from 2018 to 2022**	**Response rate**
Italy	260	870	29.89%
Greece	52	1239	4.20%
Hong Kong	12	406	2.96%
Chile	15	546	2.75%
Malaysia	10	417	2.40%
Colombia	18	851	2.12%
Portugal	12	734	1.63%
Austria	6	419	1.43%
Spain	6	425	1.41%
India	416	29834	1.39%
France	2	157	1.27%
Shanghai	3	251	1.20%
Korea	11	1143	0.96%
Taiwan (Republic of China)	5	589	0.85%
New Zealand	6	713	0.84%
Canada	35	4225	0.83%
United Arab Emirates	25	3219	0.78%
Philippines	14	1797	0.78%

Brazil	11	1467	0.75%
Germany	1	181	0.55%
Argentina	6	1900	0.32%
Ireland	1	733	0.14%
Pakistan	2	2147	0.09%
United Kingdom	3	4644	0.06%

Table S2. Wish parent survey responses.

**Affiliates**	**Number of responses**	**Total wish granted from 2018 to 2022**	**Response rate**
Italy	517	870	59.43%
Greece	248	1239	20.02%
Hong Kong	68	406	16.75%
Korea	104	1143	9.10%
Spain	32	425	7.53%
Chile	35	546	6.41%
New Zealand	36	713	5.05%
Colombia	39	851	4.58%
Malaysia	18	417	4.32%
Austria	17	419	4.06%
Canada	154	4225	3.64%
Taiwan (Republic of China)	20	589	3.40%
Portugal	19	734	2.59%
United Kingdom	106	4644	2.28%
Brazil	23	1467	1.57%

India	400	29834	1.34%
United Arab Emirates	33	3219	1.03%
Argentina	8	1900	0.42%
Australia	4	1546	0.26%
Panama	1	495	0.20%
Shanghai	8	251	0.20%
Ireland	1	733	0.14%
Philippines	2	1797	0.11%
Israel	1	1229	0.08%
